# Classification Schemes of COVID-19 High Risk Areas and Resulting Policies: A Rapid Review

**DOI:** 10.3389/fpubh.2022.769174

**Published:** 2022-02-25

**Authors:** Olalekan A. Uthman, Olatunji O. Adetokunboh, Charles Shey Wiysonge, Sameh Al-Awlaqi, Johanna Hanefeld, Charbel El Bcheraoui

**Affiliations:** ^1^Warwick Centre for Global Health Research, The University of Warwick, Coventry, United Kingdom; ^2^South African Centre for Epidemiological Modelling and Analysis, Stellenbosch University, Stellenbosch, South Africa; ^3^Division of Epidemiology and Biostatistics, Department of Global Health, Stellenbosch University, South Africa; ^4^Cochrane South Africa, South African Medical Research Council, Cape Town, South Africa; ^5^Evidence-Based Public Health, Centre for International Health Protection, Robert Koch Institute, Berlin, Germany; ^6^Centre for International Health Protection, Robert Koch Institute, Berlin, Germany

**Keywords:** COVID-19, high-risk areas, travel restrictions, travel ban, classification schemes

## Abstract

The COVID-19 pandemic has posed a significant global health threat since January 2020. Policies to reduce human mobility have been recognized to effectively control the spread of COVID-19; although the relationship between mobility, policy implementation, and virus spread remains contentious, with no clear pattern for how countries classify each other, and determine the destinations to- and from which to restrict travel. In this rapid review, we identified country classification schemes for high-risk COVID-19 areas and associated policies which mirrored the dynamic situation in 2020, with the aim of identifying any patterns that could indicate the effectiveness of such policies. We searched academic databases, including PubMed, Scopus, medRxiv, Google Scholar, and EMBASE. We also consulted web pages of the relevant government institutions in all countries. This rapid review's searches were conducted between October 2020 and December 2021. Web scraping of policy documents yielded additional 43 country reports on high-risk area classification schemes. In 43 countries from which relevant reports were identified, six issued domestic classification schemes. International classification schemes were issued by the remaining 38 countries, and these mainly used case incidence per 100,000 inhabitants as key indicator. The case incidence cut-off also varied across the countries, ranging from 20 cases per 100,000 inhabitants in the past 7 days to more than 100 cases per 100,000 inhabitants in the past 28 days. The criteria used for defining high-risk areas varied across countries, including case count, positivity rate, composite risk scores, community transmission and satisfactory laboratory testing. Countries either used case incidence in the past 7, 14 or 28 days. The resulting policies included restrictions on internal movement and international travel. The quarantine policies can be summarized into three categories: (1) 14 days self-isolation, (2) 10 days self-isolation and (3) 14 days compulsory isolation.

## Introduction

COVID19 is caused by severe acute respiratory syndrome coronavirus 2 (SARS-CoV-2) (1). As of July 1, 2021, it had infected over 182 million people worldwide and with more than 3.9 million people dead. Initial WHO recommendations regarding travel restrictions was in line with the International Health Regulation (IHR) not to restrict travel (2). Globally, responses have been swift and in full influenza pandemic control mode (3–8). Travel-related control measures comprised different interventions, including the complete closure of national borders to entry or exit, or both; full or partial travel restrictions across borders (e.g., denial of entry or exit based on the nationality, travel history, health status or other characteristics); entry and exit screening at borders based on symptoms or testing; and quarantine of travelers. These measures have been implemented for all modes of travel, including air, land, and sea (9–12).

Travel-related control measures limit the mobility of potential human carriers of infection when crossing national (and, in principle, also sub-national) borders with the aim of reducing or delaying the spread of an infectious disease across, or within countries (13, 14). In addition to implementing measures related to International Health Regulations (IHR), many countries around the world have used different national and international area-specific risk profiling schemes to inform decisions related to COVID-19 response, travels and security (2, 15–20). High risk areas classification schemes are systems that categorize countries or areas based on risk alongside the internal and international restrictions required for travel in order to protect the transmission of COVID-19.

The use of travel-related public health interventions to limit the spread of epidemic diseases has a long history. Recently, during the SARS outbreak in 2003, entry screening at national borders was deployed, and airport departure screening procedures were deployed in efforts to contain the Ebola outbreak in West Africa and the Democratic Republic of the Congo between 2014 and 2016 (21). Errett et al. (22) investigated the influence of travel restrictions on the transmission of communicable diseases other than influenza and determined that they were effective in limiting disease spread between countries but did not stop transmission. However, because the transmission properties of influenza differ from those of SARSCoV2, these findings are not directly applicable to SARSCoV2 (23, 24). Given the high rates of pre and asymptomatic transmission, certain travel-related interventions may be more appropriate than others in the SARSCoV2 pandemic (23, 24). Travel quarantine, for example, may be more effective than entry and departure screening (25).

Although the relationship between mobility, policy implementation, and virus spread remains contentious, policies to reduce human mobility have been suggested to play important role in shaping the transmission dynamics (26–29). However, evidence on how countries have classified each other during COVID-19, and how, in consequence, they have determined the destinations to- and from which to restrict travel is not systematically described. In this rapid review, we identified country classification schemes for high-risk COVID-19 areas and associated policies which mirrored the dynamic situation in 2020, with the aim of identifying any patterns that could indicate the effectiveness of such policies.

## Methods

We conducted a scoping review (30) to answer the following questions: (1) “what are the classification schemes of COVID-19 high risk areas and resulting policies?” and (2) “what are the drivers of change in classification by country?”. According to Grant and Booth (2009) (31), Scoping reviews are “preliminary assessment of potential size and scope of available research literature. Aims to identify nature and extent of research evidence (usually including ongoing research).” It has also been suggested that scoping Reviews are best designed for: “When a body of literature has not yet been comprehensively reviewed, or exhibits a large, complex, or heterogeneous nature not amenable to a more precise systematic review.” (32). Numerous scoping reviews have been conducted to examine different aspects of COVID-19 pandemic including but not limited to the role of telemedicine (33, 34), facial protection (35–37), impact on maternal and child health (38, 39), role of artificial intelligent (40–42), and estimating diagnostic accuracy of tests (43, 44).

### Eligibility Criteria

We sought to identify and characterize any published or gray literature that reported any form of COVID-19 high risk area classification schemes and travel-related control measures affecting human travel within or across national borders.

*Population:* We included studies on human populations (without any age restriction) susceptible to SARS-CoV-2/COVID-19.

*Interventions:* We included introduction and implementation any travel-related control measures affecting human travel across (international travel control policies) or within (restrictions on internal movement policies) national borders.

*Comparator(s):* We included a range of possible comparators, such as a counterfactual scenario in which the intervention was not implemented, a complete relaxation of the measure, or a partial relaxation of the measure.

*Outcome(s):* High-risk areas domestic and international classification schemes.

*Types of studies:* We considered a broad range of empirical studies whether published or gray literature that reported any form of COVID-19 high risk area classification schemes.

Studies that did not meet the inclusion criteria above were excluded.

### Information Sources and Search

The following electronic databases were searched between October 2020 and December 2021 using appropriate keywords: PubMed, Scopus, Web of Science, and medRxiv (Box 1). In addition, an advanced Google search (using the following URL: https://www.google.com/advanced_search) was implemented to identify gray literatures that are relevant to the review question. The keywords that were used for electronic database search were also applied. To conduct focused searches for all countries in Google, we combined country name with key words related to COVID-19 and high-risk areas.

Box 1 **Search terms used to identify studies addressing high-risk areas classification schemes.**

1. exp Coronavirus/
2. Coronavirus Infections/
3. COVID-19.rs.
4. severe acute respiratory syndrome coronavirus 2.os.
5. (2019 nCoV or 2019nCoV or 2019-novel CoV).ti,ab,kf.
6. (Coronavir* or corona virus* or Middle East Respiratory Syndrome* or Severe Acute Respiratory Syndrome* or SARS*).ti,ab,kf.
7. COVID 19.mp.
8. (COVID19 or COVID 2019).ti,ab,kf.
9. (nCov 2019 or nCov 19).ti,ab,kf.
10. or/1-9 [Set 1: Coronaviruses]
11. Air Travel/
12. Travel/
13. (border? adj3 (clos* or restrict* or control* or measure?)).ab,kf.
14. ((isolat* or quarantin*) adj6 (exposed or suspected or travel* or airport? or border?)).ti,ab,kf.
15. ((mobility or movement*) adj2 (reduc* or restrict*)).ti,ab,kf.
16. (travel* or border?).ti.
17. (travel adj4 (measure? or intervention? or NPI?)).ab,kf.
18. (travel* adj3 (restrict* or reduc* or control* or limit* or lockdown? or ban*)).ab,kf.
19. ((questionnaire* or screen* or surveil*) adj4 (traveler? or entr* or exit or border? or airport?)).ti,ab,kf.
20. visa?.ti,ab,kf.
21. (“unstable epidem*” OR “red list” OR “high risk area*” OR “high risk countr*” OR “high risk region”)
22. high risk areas OR high risk countries OR high risk regions

### Study Selection and Data Extraction

Two authors screened titles and abstracts of publications and websites identified (OAU and OOA). Two authors (OAU and OOA) independently charted key information from the included publications. We extracted data on the following: country, United Nations definition of region, data source (published, unpublished or policy document), policy issued date, types of high-risk areas classifications schemes (domestic classification schemes or international classification schemes), criteria used for classification scheme, resulting policies (restrictions on internal movement policies or international travel controls policies), changes in classification and drivers of changes.

### Collating, Summarizing, and Reporting the Results

Based on the primary research objectives, countries were classified into one of the following categories: type of classification scheme; criteria used for defining high-risk areas; and resulting policy types.

A detailed methodology is available Supplementary Box 1.

## Results

**Figure 1:**
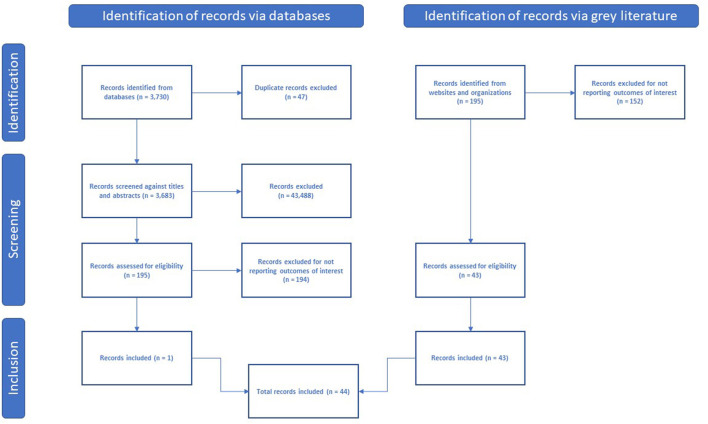
PRISMA flowchart.

The peer-reviewed literature search yielded 1,784 citations in December 2020, and 3,730 citations in December 2021. After the review of titles and abstracts, we selected 189 full-text articles for critical reading (195 later in December 2021). Only one study, conducted in Mongolia, reported a high-risk area classification scheme and met the inclusion criteria (45). The remaining 188 studies (194 later in December 2021) did not report any form of high-risk area classification scheme. Web scraping yielded policy documents from 43 countries that reported high-risk areas classification schemes. Figure 1 summarizes the search results in a PRISMA flowchart.

Summary classification schemes and resulting policies are shown in Supplementary Table 1. Most of the countries that reported classification schemes were from the European Region (*n* = 29), followed by the Americas (*n* = 7), Asia (*n* = 5), Oceania (*n* = 2), and Africa (*n* = 1). Classification schemes from 13 countries were not reported in detail. The majority of countries introduced international high-risk classification schemes (*n* = 38), while only six countries introduced domestic high-risk classification schemes. The criteria used for defining high-risk areas varied across countries and included: case count, positivity rate, composite risk scores, community transmission, and satisfactory laboratory testing. When reported, the threshold for case incidence (number of confirmed cases per 100,000 inhabitants) varied across the countries. Countries either used case incidence in the past 7 or 14 days.

### High-Risk Areas Classification Schemes

#### Domestic Classification Schemes

Domestic classification schemes were reported by six countries. Of these six, four countries [China, Kosovo, Portugal, and the United Kingdom (UK)] used case counts. The case incidence cut-off also varied across the countries, ranging from 50 cases per 100,000 in inhabitants in the past 14 days to more than 240 cases per 100,000 inhabitants in the past 14 days (Table 1). The remaining two countries were Mexico and Bolivia. Mexico used a composite risk score, while the criterion used by Bolivia is unclear. Effective from July 22, 2020, Mexico introduced four categories of risk areas (maximum, high, moderate or low) using ten epidemiologic indicators.

**Table 1:** Summary of different types of high-risk areas classification scheme.

**High-risk/red-areas classification scheme**	**Country**	**Issued date**	**Note**
**Domestic classification schemes**			
>50 infected people per 100,000 in the last 14 days	China	March 23, 2020	
*>100* infected people per 100,000 in the last 7 days	UK	November 5, 2020	Traffic light system
>151 infected people per 100,000 in the last 7 days	*Kosovo*	November 13, 2020	
*>240* infected people per 100,000 in the last 14 days	Portugal;	November 24, 2020	
**International classification schemes**			
*>20* infected people per 100,000 in the last 14 days	*Norway*	October 30, 2020	
*>25* infected people per 100,000 in the last 14 days	*Finland*	October 10, 2020	
*>40* infected people per 100,000 in the last 14 days	Slovenia	September 28, 2020	
*>50* infected people per 100,000 in the last 14 days	Estonia	October 30, 2020	
*>50* infected people per 100,000 in the last 7 days	Germany Denmark	June 19, 2020 September 25, 2020	
*>50* infected people per 100,000 in the last 28days	US CDC	November 21, 2020	
*50* to 150 infected people per 100,000 in the last 14 days	*EU*	Introduced on 13 October 2020 and amended on 28 January 2021	Traffic light system

#### International Classification Schemes

International classification schemes were reported by the remaining 38 countries. The criteria used for defining high-risk areas varied across the countries (Table 1; Supplementary Table 2). However, most used case incidence per 100,000 inhabitants. The case incidence cut-off also varied across the countries, ranging from 20 cases per 100,000 in inhabitants in the past 14 days to more than 500 cases per 100,000 inhabitants in the past 14 days (Table 1). Two countries (Grenada and Moldova) used community transmission rates to define high risk areas. Three countries (Guam, South Africa, and Cyprus) used composite risk scores to define high risk areas. The following countries did not specify criteria used for defining high risk areas: Australia, Austria, Belgium, Brunei, Canada, Malta, Montenegro, Singapore, Slovak Republic, St Vincent and the Grenadines, Trinidad and Tobago and Mongolia.

### Resulting Policies

#### Restrictions on Internal Movement Policies

**Figure 2:**
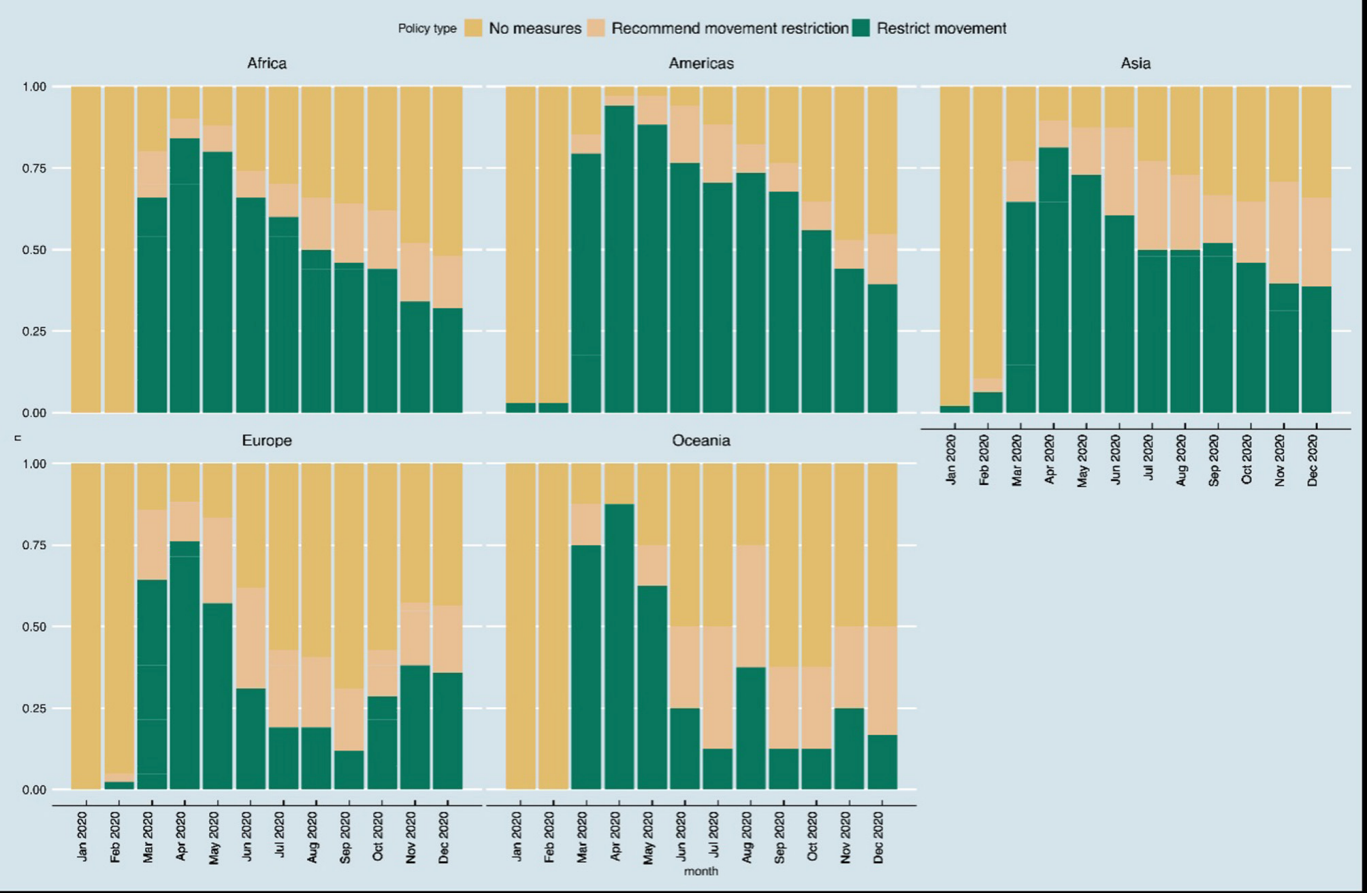
Trends in restrictions on internal movement.

**Figure 3:**
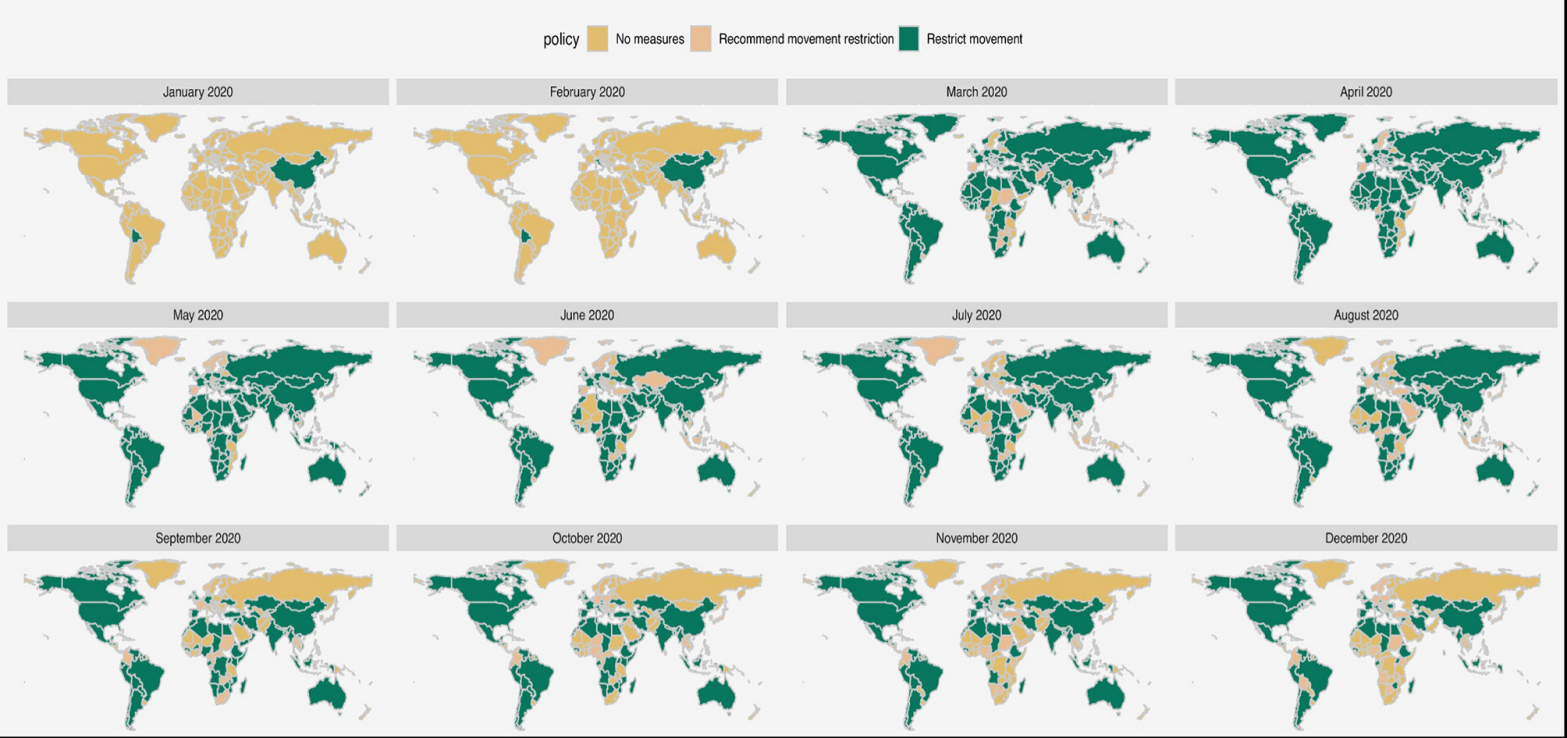
World map of trends in restrictions on internal movement.

The first internal movement policies due to COVID-19 were issued between January 1, 2020 in Bolivia and November 30, 2020 in Hong Kong (Supplementary Table 3). The trends in different types of restrictions on internal movement as collated by Oxford COVID-19 Government Response Tracker are shown in Figures 2, 3. None of the African countries instituted any restrictions on internal movement between January and February 2020. However, between March 2020 and August 2020, more than 50% of the countries restricted internal movement. The trends on pattern of restrictions on internal movement were similar among countries from the Americas and Asia. In Europe and Oceania, restrictions on internal movement were relaxed in many countries starting June 2020.

#### International Travel Controls Policies

**Figure 4:**
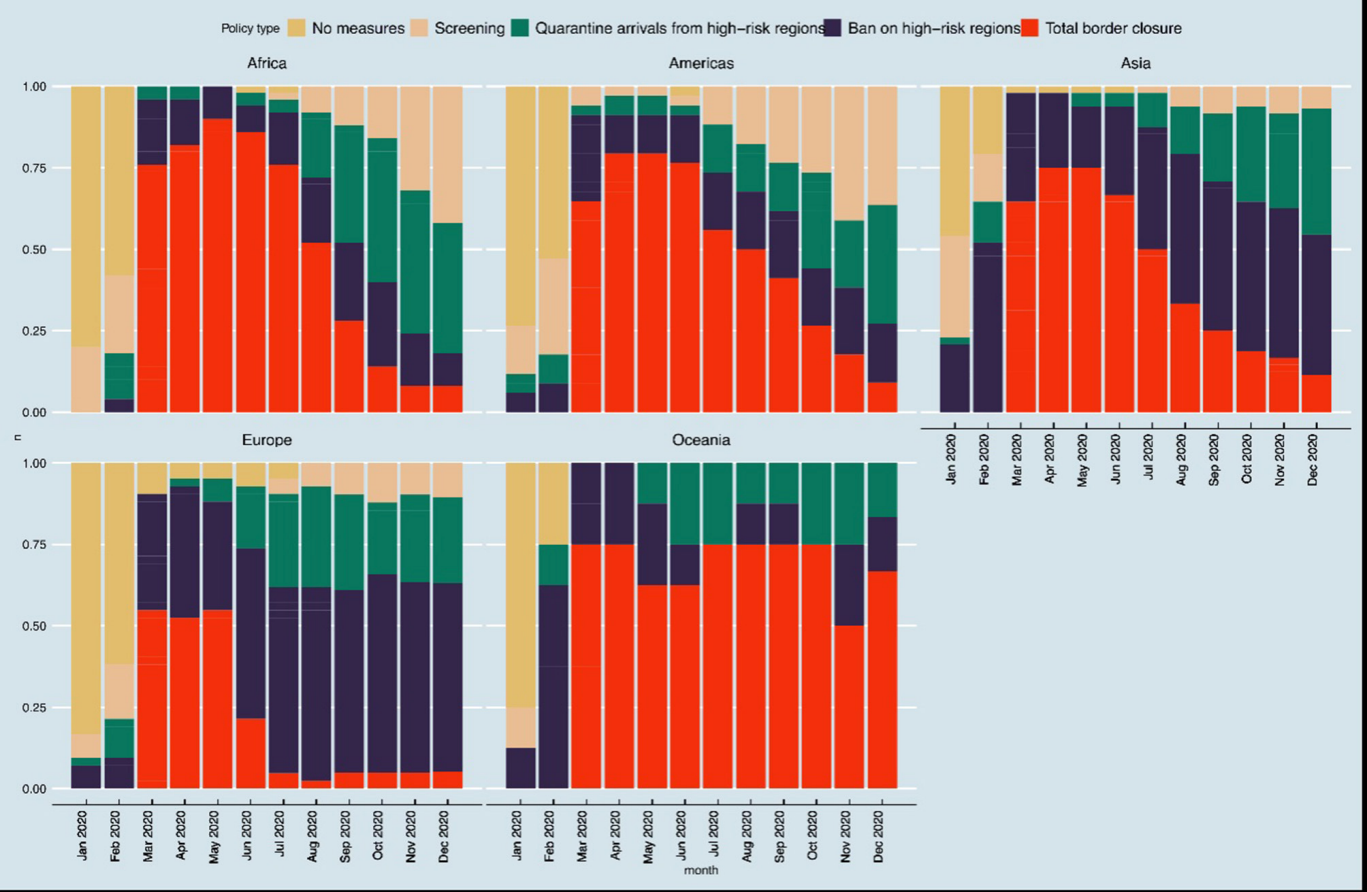
Trends in international travel controls policies.

**Figure 5:**
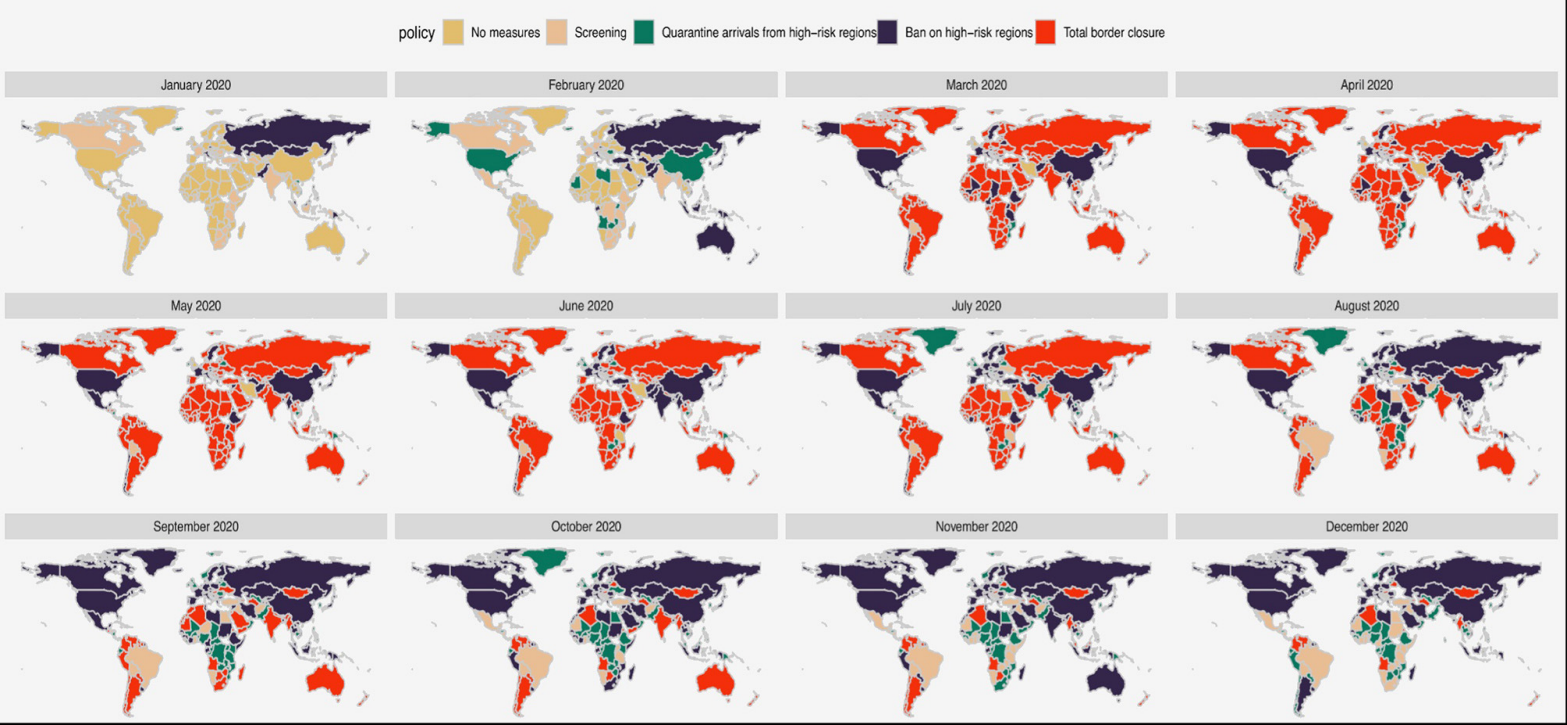
World map of trends in international travel controls policies.

The first international travel controls policies due to COVID-19 were issued between January 1, 2020 in Bolivia, Hong Kong, and Taiwan and August 12, 2020 in Luxemburg (Supplementary Table 3). The trends in different types of international travel control policies as collated by Oxford COVID-19 Government Response Tracker (46) are shown in Figures 4, 5. The trends in pattern of international travel controls were similar among countries from Africa, the Americas and Asia. In Africa, 10 of the 50 countries instituted ban on high-risk regions (20%), while 38 of the 50 countries (76%) instituted total border closure after the COVID-19 was declared a pandemic from March 2020. African countries started relaxing the total border closure by August 2020 and by November 2020, only 4 of the 50 countries (8%) in Africa still instituted total border closure. In the Americas, 27 of the 34 countries (79%) instituted total border closure after the COVID-19 in April 2020; and similarly, 36 of the 48 Asian countries (75%) instituted total border closure after the COVID-19 in April 2020. While in Europe, 23 of the 42 countries (55%) instituted total border closure after the COVID-19 was declared a pandemic from March 2020. However, European countries started relaxing the total border closure earlier than other regions, by June 2020, when only 9 of the 42 countries (21%) still instituted total border closure.

The type of quarantine policies varied across the countries. Table 2 summarized different types of isolation and quarantine policies. The quarantine policies can be summarized into three categories: (1) 14 days self-isolation (Channel Islands of Jersey, Cyprus, Denmark, Slovenia, Ireland, Italy, Australia, Montenegro, Trinidad and Tobago and Moldova, Iceland, Brunei, St Vincent, and the Grenadines); (2) 10 days self-isolation (Austria, Finland, Norway, Latvia, Liechtenstein, Lithuania, Luxemburg, and Slovak Republic); and (3) 14 days compulsory isolation (China, Malta, Guam, Mongolia, Grenada).

**Table 2:** Summary of different types of isolation and quarantine policies.

**Policy**	**Country**
14 days self-isolation	Channel Islands of Jersey, Cyprus, Denmark, Slovenia, Ireland, Italy, Australia, Montenegro, Trinidad and Tobago and Moldova, Iceland, Brunei, St Vincent and the Grenadines
14 days compulsory isolation	China, Malta, Guam, Mongolia, Grenada
10 days self-isolation	Austria, Finland, Norway, Latvia, Liechtenstein, Lithuania, Luxemburg and Slovak Republic

## Discussion

We show in this scoping review that many countries adopted different classification schemes for COVID-19 high-risk areas and resulting policies since the beginning of the COVID-19 outbreak. The high-risk COVID-19 areas and associated policies were based on COVID-19 cases estimated to be possibly avoided, number of cases detected and shift in epidemic development due to interventions. Classification schemes for some of the countries were not reported in detail. Most countries in the European Union and North America formulated well-defined high-risk areas classification schemes and travel restriction regulations.

On January 30, 2020, the WHO Director-General declared COVID-19 outbreak a public health emergency of international concern, prompting many countries to adopt international and domestic travel restrictions to prevent importation of COVID-19 infections (47) *even before WHO* upgrading their declaration to a COVID-19 pandemic on March 11, 2020 (48).

First, countries needed classification schemes for other countries, and for their own geographic organization, to determine the risk of international and domestic travel, and consequent measures. We documented classification schemes of COVID-19 high-risk areas and resulting policies either as they relate to international travel or domestic movement for 43 countries. These were issued between March and November 2020. Most of the 43 countries used case counts as the main criteria for classifying high-risk areas. It has been well-reported previously that apparent case rates are a function of testing rates. Stephen and colleagues (49) have' cautioned that policy should not be based on such a metric which is also a function of government policy. Alternative or combined measures should be sought to avoid creating perverse incentives in policy development e.g., penalizing countries with the best testing regimen, test positivity rates are one such attempt at this. Imposing travel restrictions on countries with apparent high case rates could create a perverse incentive for such countries to test less, or to not publicly report testing rates. It may not be plausible for any responsible government to deliberately reduce testing, but government commitment to testing does vary by country for practical, financial, and political reasons. Hence, penalizing countries with high apparent rates could provide a disincentive to the roll-out of greater testing, an essential component of pandemic control which proved to be specifically helpful during this first wave of the COVID-19 pandemic (50). Further, how countries develop their classification schemes is also a matter of their political environment and connected to the nature of their response in terms of how assertive it is (51). For instance, a more assertive response generates higher trust, and vice-versa (52).

While the COVID-19 pandemic is highly dynamic, with a wide spectrum of epidemiological variability by country, risk area classification schemes can by their nature not be adapted at the same pace as the pandemic, or in conjunction with the epidemiological variability, by all countries. Some of the countries like Guam and Mexico did not update the high-risk areas classification schemes concerning travels from different destinations for weeks. Placing destination countries on the same level of risk estimation for many weeks despite changes in the number of cases and positivity rates shows non-adoption of data-driven, evidence-based, and scientific approaches to pandemic control. Most countries in the European Union adopted highly dynamic classification schemes that reflected more closely the changes in the pandemic. In parallel, countries also adopted a wide variety of travel policies, mostly divided into border closure, quarantine and screening travelers (53). Very few countries imposed total lockdowns, an intervention avoided for many reasons despite its big potential to reduce infections and deaths by up to 75% and 38 (54). Further to what can be feasible, travel policies should be in tune with changes in the epidemiological trend of COVID-19. What we've learned though is that the timeliness of implementing such policies, specifically at the beginning of the outbreak, and the degree of compliance, are major factors to their effectiveness (53). At the early stage of the outbreak, travelers coming from high burden countries may import cases and contribute to the local COVID-19 burden (55). The effect of non-restrictive travel may be pronounced in countries or regions with low COVID-19 infection burden and those at the receiving end of a large volume of arrivals from high burden countries (55).

We found that as much as 38 of the 50 countries instituted total border closure after the COVID-19 was declared a pandemic from March 2020; and started relaxing the total border closure by August 2020 and by November 2020. While most of the countries in the Americas instituted total border closure after the COVID-19 in April 2020; and similarly, 36 of the 48 Asian countries instituted total border closure after the COVID-19 in April 2020. While in Europe, only about 23 of the 42 countries instituted total border closure after the COVID-19 was declared a pandemic from March 2020. In addition, European countries started relaxing the total border closure earlier than other regions, by June 2020, when only 9 of the 42 countries still instituted total border closure. The degree to which border closure has worked varied by context. Nevertheless, border closure is not enough if not coupled with other physical distancing policies (53). Indeed, a study comparing three Southeast Asian countries during the COVID-19 pandemic shows a relatively higher success of Singapore in controlling the number of cases, as well as fatality rate due to national lockdown and a stronger health system (56).

Virtually every country implemented some form of travel restrictions, however, our findings and a study by Habibi et al. indicated that some of the high-risk areas' classification schemes were developed and implemented without using comprehensive evidence-based criteria (18, 57). Countries that implemented total border closures could have tried to assess how selective restriction policies could have had played. For instance, such countries that welcome non-stationary laborers from neighboring countries could have controlled and monitored this flow more closely to strategically plan for similar scenarios in the future. Developing policies for a new disease like COVID-19 requires evidence-based approaches that will include the use of the best available facts and materials that are specific for similar novel infections and with clearly defined outcomes of interest (57). Further, the decision-making process in selecting the policies to be implemented differs greatly between countries. While investigating such process is outside the scope of this work, we note how countries differ I what public health agencies they refer to, whether national or regional. The United States CDC used both primary and secondary criteria to determine different destinations' Travel Health Notice (THN) levels (58) while countries within the European Union/European Economic Area implemented the ECDC guidelines (59). The EU Recommendation on a Structured Approach to Travel Restrictions in the Form of COVID-19 was adopted by Member States on October 13th. This “traffic light” approach categorizes regions in the European Union (EU) and European Economic Area (EEA) as green, orange, red, or gray based on the risk levels associated with COVID-19 (59). On January 28, 2021, a new dark red category was added to the weekly published map for areas where the COVID-19 virus is circulating at very high levels, including due to more infectious variants (59). Aleta et al. show that travel restrictions are likely to be effective measures only in the short term and may be less effective at a later stage and for the elimination of the infection (15, 60). In order to have a better control of COVID-19 pandemic, other preventive and control measures such as promotion of the use of face masks, regular handwashing, school closures, and suspension of large public get-togethers should also be place (61). Other measures such as active surveillance and self-isolation of infected persons and their contacts should also be implemented (45).

We found one recent Cochrane review on “International travel-related control measures to contain the COVID-19 pandemic: a rapid review” (25). The review includes 62 studies conducted around the world and at various stages of the pandemic (25). Most of the studies included in this review compared existing travel-related control measures to no restrictions at all (25). Most of the studies found that travel restrictions that reduced or stopped cross-border movement were helpful, though the magnitude of this benefit varied. Furthermore, several studies found no effect (25). Findings from modeling studies showed that 1–53% of cases can be detected. In addition, testing travelers could lower the number of cases imported or exported, as well as cases discovered. The review also reported that quarantine appeared to be beneficial in all the studies, however the magnitude of this benefit varied from small to large effect. Most studies found some benefit from quarantine and border screening. Effects may vary depending on how long they were quarantined and how often they were tested throughout that time. The review concluded that overall, international travel-related control measures, may help to prevent the spread of COVID19 across national borders (25). Cross-border travel restrictions can be beneficial. Travelers who are merely screened for symptoms at borders are likely to miss many cases; testing, while more successful, may also miss cases if done immediately after arrival. A 10-day quarantine can help prevent the spread of COVID-19, and it may be more successful when combined with another intervention like testing, especially if people follow the restrictions (25).

This review has several strengths. Comprehensive searches of several databases and gray literature sources were conducted to identify eligible articles and documents that will result in the highest quality of evidence. Two reviewers independently screened the search outputs and extracted data from included documents. At the same time, this review has some limitations. First, we set out to include all types of publications especially peer-reviewed papers, and to compare the effectiveness of the different classification schemes on the epidemiological evolution. However, only one of the included documents was a peer-reviewed published paper. The systematic search for peer-reviewed literature did not yield the desired outputs and the included documents were found *via* random Google searches of several governmental, policy, and travel sites for more than 200 countries and territories. Second, some of the materials were not in the English language and warranted an additional translation process.

Third, some of the official websites on travel restrictions do not keep historic versions of their policies and were not updated, thereby restricting accurate timeline development, as well as policy change analysis. In addition, it was not possible to access the steps or how the countries choose the criteria used for defining high risk areas. Based on the available evidence it was not possible to access the link between types of classification schemes and resulting policies. Formal modeling of the impact of types of classification schemes and resulting policies on the COVID-19 epidemic should be considered.

## Conclusion

There was substantial variation between and within countries in the measures that governments adopted and how quickly they have adopted them in classifying high-risk areas. In 43 countries from which relevant reports were identified, six issues domestic classification schemes. International classification schemes were issued by the remaining 38 countries and mainly used case incidence per 100,000 inhabitants. The case incidence cut-off also varied across the countries, ranging from 20 cases per 100,000 in inhabitants in the past 7 days to more than 100 cases per 100,000 inhabitants in the past 28 days. The resulting policies included restrictions on internal movement and international travel control. The quarantine policies can be summarized into three categories: (1) 14 days self-isolation, (2) 10 days self-isolation and (3) 14 days compulsory isolation.

## Data Availability Statement

The original contributions presented in the study are included in the article/Supplementary Material, further inquiries can be directed to the corresponding authors.

## Author Contributions

OU, OA, CW, SA-A, JH, and CE were involved in the conception of the study. OU and OA carried out the searches, data extraction and drafted the paper with contributions from all authors. All authors read and approved the final manuscript.

## Funding

This research was commissioned by the Center for International Health Protection, Robert Koch Institute.

## Author Disclaimer

The views expressed in this publication are those of the authors and not necessarily those of the Robert Koch Institute.

## Conflict of Interest

The authors declare that the research was conducted in the absence of any commercial or financial relationships that could be construed as a potential conflict of interest.

## Publisher's Note

All claims expressed in this article are solely those of the authors and do not necessarily represent those of their affiliated organizations, or those of the publisher, the editors and the reviewers. Any product that may be evaluated in this article, or claim that may be made by its manufacturer, is not guaranteed or endorsed by the publisher.
