# Supplementary material for: Classification Schemes of COVID-19 High Risk Areas and Resulting Policies: A Rapid Review
Source: Front Public Health. 2022 Feb 25;10:769174. doi: 10.3389/fpubh.2022.769174 (PMC8916531; doi:10.3389/fpubh.2022.769174)
Supplement: Supplementary file 1 [file Table_1.DOCX]

Supplementary Material

**Box 1: Detailed Methods**

| - **Stage 1: Identify the research question**   The review questions were: (1) “what are the classification schemes of COVID-19 high risk areas and resulting policies?” and (2) “what are the drivers of change in classification by country?”.   - **Stage 2: Identifying relevant studies**   This rapid review’s searches were conducted between October 7 and December 31, 2020. We formulated a comprehensive and exhaustive search strategy in order to identify all relevant studies regardless of language or publication status (published, unpublished, in press, and in progress) appropriate for answering the research questions. The following electronic databases were searched using appropriate keywords: PubMed, Scopus, Web of Science, and medRxiv (Box 1). In addition, an advanced Google search (using the following URL: https://www.google.com/advanced_search) was implemented to identify grey literatures that are relevant to the review question. The keywords that were used for electronic database search were also applied.  The search strategy was structured around two blocks focusing on (1) COVID‐19, severe acute respiratory syndrome (SARS) and (2) travel‐related control measures. We conducted the searches in English and included studies published in other languages (see “Language” above). Critical keywords and thesaurus heading terms were initially tailored to PubMed searches and then adapted for other sources as necessary (Box 2). To conduct focused searches for all countries in Google, we combined country name with key words related to COVID-19 and high-risk areas.   - **Stage 3: Study selection**   A set of eligibility criteria were developed while preparing the protocol, specifying the types of eligible studies, participants, and outcomes.  Types of studies: We considered a broad range of empirical studies whether published or grey literature that reported any form of COVID-19 high risk area classification schemes.  Types of participants: We included human populations (without any age restriction) susceptible to human coronavirus diseases, namely severe acute respiratory syndrome coronavirus 1(SARS‐CoV‐1)/SARS, SARS‐CoV‐2/COVID‐19.  Types of interventions: We considered travel‐related control measures affecting human travel within or across national borders.  Types of outcomes: Classification schemes for high-risk areas   - **Stage 4: Charting the data**   Two authors independently charted key information from the included publications. An Excel spreadsheet was used for this purpose. Using a pre-tested data extraction form, two reviewers independently extracted data from included studies. We extracted data on the following: country, United Nations definition of region, data source (published, unpublished or policy document), policy issued date, types of high-risk areas classifications schemes (domestic classification schemes or international classification schemes), criteria used for classification scheme, resulting policies (restrictions on internal movement policies or international travel controls policies), changes in classification and drivers of changes.    - **Stage 5: Collating, summarizing, and reporting the results**   Based on the primary research objectives, countries were classified into one of the following categories: type of classification scheme (domestic versus international classification schemes); criteria used for defining high-risk areas (epidemiological data versus composite risk scores); and resulting policy types (quarantine policy and travel restrictions). The travel restriction policies were further categorized into two: restrictions on internal movement (no measures, recommended restriction or restrict movement) and international travel controls (no measures, screening, quarantine on arrival from high-risk regions, ban on travel from high-risk regions, or total border closure). Due to the nature of the research questions, it was not possible to synthesize the results quantitatively. We thus decided to use a narrative synthesis approach to present details for each country and discuss them in turn. For the trend data in both restrictions on internal movement and international travel controls, agglomerative hierarchical clustering was used to explore the data and we used elbow method[7] to determine the ideal number of clusters for each region. In addition, we also ‘manually’ clustered the data based on the type of criteria used for classifying high-risk areas. |
| --- |

**Supplementary Table 1: Summary classification schemes and resulting policies**

| **Country** | **United Nation Region** | **Type of classification scheme** | **Criteria Type** | **High risk definition** | **Policies: Restrictions** |
| --- | --- | --- | --- | --- | --- |
| China | Asia | Domestic classification scheme | Case count | Low-risk areas – are areas with no confirmed cases or no new confirmed cases for 14 consecutive days; Medium-risk areas – are those with new confirmed cases within 14 days – but the total new cases are no more than 50,or with cumulatively more than 50 confirmed cases – but no cluster epidemic within 14 days; and High-risk areas – are those where the cumulative number of confirmed cases have exceeded 50 cases, and a cluster epidemic was recorded within the last 14 days. | Travelers will be subject to a 14-day mandatory quarantine in the first entry point city. After they complete the fortnight quarantines and test negative for the virus, if they proceed to Beijing on the same or following day, they will not need to undergo another 14-day quarantine in Beijing |
| Kosovo | Europe | Domestic classification scheme | Case count | The level of restrictions varies by municipality, split into three categories of risk.  The red zone includes high risk municipalities or municipalities with over 151 infected per 100,000 inhabitants per week.  The yellow zone includes municipalities with medium risk, or municipalities from 76 to 150 infected per 100,000 inhabitants per week.   The green area includes low risk municipalities or municipalities from 1 to 75 infected per 100,000 inhabitants per week. | Red zone – high risk All restaurants, bars, lounges and pubs under this label must stop their activity by 19:00 until 05:00 and citizens or vehicles are not allowed to go out of the house, except emergency cases.  Yellow zone – medium risk Municipalities under this label are allowed to keep businesses open from 05:00 to 18:00. Public transport is allowed to operate at 50% capacity.  Green zone – low risk The green zone includes municipalities of Kosovo with a low risk of infection. Businesses in these municipalities will be allowed to stay open from 05:00 to 20:00. |
| Portugal | Europe | Domestic classification scheme | Case count | Update the list of municipalities with a high risk of contagion. The measures to combat Covid-19 will be applied depending on the epidemiological situation in each municipality. Thus, and following the criteria determined by the European Center for Disease Prevention and Control (ECDC), four levels of severity of the pandemic were distinguished : - Moderate : Municipalities with less than 240 cases per 100 thousand inhabitants in the last 14 days; - High : Municipalities with a number of cases between 240 and 479 per 100 thousand inhabitants in the last 14 days; - Very high : Municipalities with a number of cases between 480 and 959 per 100 thousand inhabitants in the last 14 days; - Extremely high : Municipalities with more than 960 cases per 100 thousand inhabitants in the last 14 days. | '- For the entire continental territory : - Prohibition of movement between municipalities in the following periods:  - Between 11 pm on 27 November and 5 am on 2 December;  - Between 11 pm on December 4 and 5 am on December 9;  - Time tolerance and suspension of teaching activity and call for the dismissal of private sector workers on November 30 and December 7; - Mandatory use of a mask in the workplace.  -  - For municipalities with a “ high ” risk level , in addition to the measures applied to the entire continental territory:  - Prohibition of circulation on public roads between 11 pm and 5 am;  - Action to monitor compliance with mandatory teleworking ; - Maintenance of the opening hours of the establishments (10 pm, except restaurants and cultural facilities at 10:30 pm).  -  - For “ very high ” and “ extremely high ” municipalities , in addition to the measures applied to the entire national territory, the following also apply:  - Prohibition of circulation on public roads between 11 pm and 5 am on weekdays ;  - Prohibition of circulation on public roads on Saturdays and Sundays between 1 pm and 5 am;  - Prohibition of circulation on public roads on December 1 and 8 between 1 pm and 5 am;  - On November 30 and December 7 , commercial establishments must close at 3 pm ;  - Action to monitor compliance with mandatory teleworking ; |
| United Kingdom | Europe | Domestic classification scheme | Case count | Tier 1 ‘Medium’: Infection rate: fewer than 100 cases per 100,000 people. Tier 2 ‘High’: Infection rate: over 100 cases per 100,000 people Tier 3 – ‘Very High’: Tier 3 will be enforced where the rate of transmission has not reduced despite the introduction of measures under Tier 2. | Tier 1 - Medium Alert:  There are no restrictions on travel or use of transport but you should still wear a face covering.  Avoid travelling to tier three areas unless where necessary for example for work, medical reasons, caring or education.    If you do travel to a tier 2 or tier 3 area you will need to follow the rules of that area while you are there.  Tier 2 - High Alert: Journeys should be limited where possible, but you can still travel and use transport to go to the shops, work and hospitality venues that are open. You should still wear a face covering.  Avoid travelling to tier 3 areas unless where necessary for example for work, medical reasons, caring or education.   If you travel into a tier 3 area then you will need to follow the rules of tier 3.    Tier 3 - Very High Alert: Journeys should be minimized, but you can still travel to go to the shops, works and hospitality venues that are open. You should still wear a face covering.  You're advised against travelling out of your area (although exceptions apply, for example for work, education or caring responsibilities). |
| Bolivia | Americas | Domestic classification scheme | Not specified | Towns and cities are classified to be at high, medium or moderate risk based on coronavirus data. |  |
| Mexico | Americas | Domestic classification scheme | Composite risk score | COVID-19 epidemic risk - Traffic light, ten indicators were considered that represent transmission between people, territorial spread, response capacity, the consequences of the epidemic on health and life. | Mexican embassies and consulates around the world have suspended operations and visa issuance (including for visitors and temporary residents). Issuance of national passports suspended, in an effort to prevent citizens from traveling abroad. Delays in application processing (and in some offices, limits to number of applications accepted each day) expected by the National Migration Institute, where there is reduced personnel. |
| Channel Islands of Jersey | Europe | International classification scheme | Case count | This daily case data is then used to calculate a 14-day case notification rate per 100,000 population for the most recent 14-day period for which data is available. Areas are then categorized as: - Green if the rate is below 50 per 100,000 - Amber if the rate is between 50 per 100,000 and 120 per 100,000 - Red if the rate is over 120 per 100,000 The 14 day case notification rate is calculated by summing the number of daily cases in an area in the past 14 days and dividing this number by the total population that live in that area. This is then multiplied by 100,000 to allow comparisons between areas of different sizes. 14-day rates are not comparable to 7-day rates. | All passengers are required to isolate for 14 days unless given permission not to do so. |
| Denmark | Europe | International classification scheme | Case count | Travelers from regions where the infection rate is above 50 new infections per 100,000 inhabitants per week, are advised to be tested upon arrival. Andorra, Malta, Slovenia, Iceland, Ireland, and the United Kingdom are classified as banned countries. | Travelers are advised to self-quarantine for 14 days upon their return to Denmark from a country not included in the list of open countries. |
| Estonia | Europe | International classification scheme | Case count | a 10-day quarantine obligation has been in force for entry from countries whose coefficient of infection is above 50 new infections per 100,000 inhabitants in a two-week period. | Travelers from listed countries can alternatively be tested for COVID-19 upon entry and must wait for the negative result in self-isolation. Afterwards they can go to work with limited social contacts. After seven days at the earliest, a second test must take place. |
| Finland | Europe | International classification scheme | Case count | The Government will reassess the need for and extent of the restrictions on entry once a week based on the epidemiological situation. When entry from a certain country is restricted due to a high incidence of COVID-19, recreational travel to Finland from that country is not permitted. In this case, only return traffic to Finland, transit traffic, commuting or travel for other essential reasons is possible. Self-isolation and testing are recommended for travelers arriving in Finland from high-incidence countries.  Any changes to the restrictions will be made on a weekly basis by the Ministry of the Interior based on an expert evaluation by the Finnish Institute for Health and Welfare. The limit value of 25 new cases per 100,000 persons in the previous 14 days will apply to the EU and Schengen countries and the countries on the Green List defined by the Council of the European Union. The changes will be decided on at the Thursday government session. | People may still come to Finland from the following European countries for work or other essential reasons: Andorra, Bulgaria, Croatia, Cyprus, Ireland, Monaco, Romania, San Marino and the United Kingdom. The restrictions on entry for these countries correspond to the restrictions on internal border traffic. A 10-day period of self-isolation is recommended for travelers arriving in Finland. |
| Germany | Europe | International classification scheme | Case count | A country or an area is defined as "high-risk" when there are more than 50 new infections per 100,000 people over the last seven days. | Travelers entering Germany, who have been in a risk area at any time within the 14 days prior their entry, are obliged to self-isolate or to present a negative COVID-19 test result in accordance with the respective quarantine regulations of the responsible federal state. |
| Norway | Europe | International classification scheme | Case count | The requirements for quarantine do not apply to travelers who are resident in countries in the EU/EEA/Schengen area with fewer than 20 cases per 100,000 inhabitants during the last two weeks, and fewer than 5% positive tests on average per week over the last two weeks. | If travelers arrive from a country or an area that are defined as high transmission areas, they must go into quarantine for 10 days from the day they arrive in Norway. For travelers from a "red" or "striped" area, a 10-day quarantine is mandatory. Currently, no countries are marked as green. |
| Slovenia | Europe | International classification scheme | Case count | The red list of epidemiologically countries includes such countries which registered more than 40 cases per 100,000 inhabitants over two weeks. | Permanent or temporary residents from countries that are on the list of epidemiologically countries, require a 14-day quarantine. |
| South Africa | Africa | International classification scheme | Case count | High-risk travelers are those who come from countries with higher numbers of COVID-19 infections and reported deaths compared to South Africa.  Medium risk travelers are from countries with a relatively equal number of infections and death toll to South Africa and low-risk travelers originate from countries with lesser number of infections of COVID-19 and death toll than South Africa.  Low-risk travelers originate from countries with lesser number of infections of COVID-19 and death toll than South Africa. |  |
| United States of America | Americas | International classification scheme | Case count | Primary criteria for destinations with populations over 200,000 1. Incidence rate (cumulative new cases per 100,000 people over the past 28 days) 2. New case trajectory (Are new cases over the past 28 days increasing, decreasing, or stable?) LEVEL 4 - VERY HIGH Level: >100 LEVEL 3 - HIGH Level: 51 - 100  LEVEL 2 - MODERATE Level: 5-50 LEVEL 1 - LOW Level: <5   Primary criteria for destinations with a population of 200,000 or less 1. COVID-19 case counts (cumulative new cases over the past 28 days) 2. New case trajectory (Are new cases over the past 28 days increasing, decreasing, or stable?) LEVEL 4 - VERY HIGH Level: >100 LEVEL 3 - HIGH Level: 51 - 100 LEVEL 2 - MODERATE Level: 10-50 LEVEL 1 - LOW Level: <10    Secondary Criteria CDC uses hospitalization rates and cumulative testing positivity rate as secondary criteria to validate the primary criteria. Both primary and secondary criteria are measured over 28 days. Secondary criteria data are obtained from official sources, such as ministry of health websites. CDC reviews secondary criteria for all destinations, regardless |  |
| Croatia | Europe | International classification scheme | Case count, Test positivity rate | EU Traffic Lights Approach to Travel  Green if the 14-day notification rate is lower than 25 cases per 100 000 and the test positivity rate below 4%;  Orange if the 14-day notification rate is lower than 50 cases per 100 000 but the test positivity rate is 4% or higher or, if the 14-day notification rate is between 25 and 150 cases per 100 000 and the test positivity rate is below 4%;  Red if the 14-day notification rate is 50 cases per 100 000 or higher and the test positivity rate is 4% or higher or if the 14-day notification rate is higher than 150 cases per 100 000;  Grey if there is insufficient information or if the testing rate is lower than 300 cases per 100 000. |  |
| Czech Republic | Europe | International classification scheme | Case count, Test positivity rate | EU Traffic Lights Approach to Travel  Green if the 14-day notification rate is lower than 25 cases per 100 000 and the test positivity rate below 4%;  Orange if the 14-day notification rate is lower than 50 cases per 100 000 but the test positivity rate is 4% or higher or, if the 14-day notification rate is between 25 and 150 cases per 100 000 and the test positivity rate is below 4%;  Red if the 14-day notification rate is 50 cases per 100 000 or higher and the test positivity rate is 4% or higher or if the 14-day notification rate is higher than 150 cases per 100 000;  Grey if there is insufficient information or if the testing rate is lower than 300 cases per 100 000. | For non-EU countries classified as "high-risk" (red countries), it is mandatory to present a negative test for COVID-19 to the regional hygienic station within five days from the entry. Furthermore, a Passenger Locator Form (https://plf.uzis.cz/) is mandatory. The above-mentioned requirements are not necessary if your stay in a red country did not exceed 12 hours in the last 14 days, for cross-border workers, pupils and students coming from neighboring countries; in case of urgent travel (for work and/or health-related reasons) for a period of time not exceeding 12 hours; or in case of transit up to 12 hours. A test carried out in another EU country not older than 72 hours can be submitted to the local hygiene institute immediately after entry. Until the test result is presented, there is a quarantine obligation. |
| Hungary | Europe | International classification scheme | Case count, Test positivity rate | EU Traffic Lights Approach to Travel  Green if the 14-day notification rate is lower than 25 cases per 100 000 and the test positivity rate below 4%;  Orange if the 14-day notification rate is lower than 50 cases per 100 000 but the test positivity rate is 4% or higher or, if the 14-day notification rate is between 25 and 150 cases per 100 000 and the test positivity rate is below 4%;  Red if the 14-day notification rate is 50 cases per 100 000 or higher and the test positivity rate is 4% or higher or if the 14-day notification rate is higher than 150 cases per 100 000;  Grey if there is insufficient information or if the testing rate is lower than 300 cases per 100 000. | Travelers that can enter with permission​​n must self-quarantine. Foreign students must evidence COVID-19 tests with a negative result unless they are allowed to study. |
| Iceland | Europe | International classification scheme | Case count, Test positivity rate | EU Traffic Lights Approach to Travel  Green if the 14-day notification rate is lower than 25 cases per 100 000 and the test positivity rate below 4%;  Orange if the 14-day notification rate is lower than 50 cases per 100 000 but the test positivity rate is 4% or higher or, if the 14-day notification rate is between 25 and 150 cases per 100 000 and the test positivity rate is below 4%;  Red if the 14-day notification rate is 50 cases per 100 000 or higher and the test positivity rate is 4% or higher or if the 14-day notification rate is higher than 150 cases per 100 000;  Grey if there is insufficient information or if the testing rate is lower than 300 cases per 100 000. | All passengers born before 2005 arriving in Iceland must pre-register in order to be screened for COVID-19. The government of Iceland has implemented more comprehensive border-screening measures as of August 19, 2020. All travelers arriving in Iceland must choose between undergoing a quarantine of 14 days or participating in a double screening measure, including a 5-6 days quarantine until the results of the second test are known. If the second PCR-test is negative no further measures are required. Those who test positive are required to self-isolate. Children born in 2005 or later are exempt from the double border-screening procedure. |
| Ireland | Europe | International classification scheme | Case count, Test positivity rate | EU Traffic Lights Approach to Travel  Green if the 14-day notification rate is lower than 25 cases per 100 000 and the test positivity rate below 4%;  Orange if the 14-day notification rate is lower than 50 cases per 100 000 but the test positivity rate is 4% or higher or, if the 14-day notification rate is between 25 and 150 cases per 100 000 and the test positivity rate is below 4%;  Red if the 14-day notification rate is 50 cases per 100 000 or higher and the test positivity rate is 4% or higher or if the 14-day notification rate is higher than 150 cases per 100 000;  Grey if there is insufficient information or if the testing rate is lower than 300 cases per 100 000. | Passengers arriving from territories that are not included on the” green list” are expected to self-isolate for 14 days. |
| Italy | Europe | International classification scheme | Case count, Test positivity rate | EU Traffic Lights Approach to Travel  Green if the 14-day notification rate is lower than 25 cases per 100 000 and the test positivity rate below 4%;  Orange if the 14-day notification rate is lower than 50 cases per 100 000 but the test positivity rate is 4% or higher or, if the 14-day notification rate is between 25 and 150 cases per 100 000 and the test positivity rate is below 4%;  Red if the 14-day notification rate is 50 cases per 100 000 or higher and the test positivity rate is 4% or higher or if the 14-day notification rate is higher than 150 cases per 100 000;  Grey if there is insufficient information or if the testing rate is lower than 300 cases per 100 000. | On entering Italy, they must self-isolate and undergo supervision by the competent health authorities for 14 days​. |
| Latvia | Europe | International classification scheme | Case count, Test positivity rate | EU Traffic Lights Approach to Travel  Green if the 14-day notification rate is lower than 25 cases per 100 000 and the test positivity rate below 4%;  Orange if the 14-day notification rate is lower than 50 cases per 100 000 but the test positivity rate is 4% or higher or, if the 14-day notification rate is between 25 and 150 cases per 100 000 and the test positivity rate is below 4%;  Red if the 14-day notification rate is 50 cases per 100 000 or higher and the test positivity rate is 4% or higher or if the 14-day notification rate is higher than 150 cases per 100 000;  Grey if there is insufficient information or if the testing rate is lower than 300 cases per 100 000. | All travelers, including citizens and permanent residents of Latvia, who have arrived from countries in which the 14-day cumulative number of COVID-19 cases has exceeded a specific defined number of cases per 100,000 inhabitants. Must self-isolate for a 10-day period upon arrival |
| Liechtenstein | Europe | International classification scheme | Case count, Test positivity rate | EU Traffic Lights Approach to Travel  Green if the 14-day notification rate is lower than 25 cases per 100 000 and the test positivity rate below 4%;  Orange if the 14-day notification rate is lower than 50 cases per 100 000 but the test positivity rate is 4% or higher or, if the 14-day notification rate is between 25 and 150 cases per 100 000 and the test positivity rate is below 4%;  Red if the 14-day notification rate is 50 cases per 100 000 or higher and the test positivity rate is 4% or higher or if the 14-day notification rate is higher than 150 cases per 100 000;  Grey if there is insufficient information or if the testing rate is lower than 300 cases per 100 000. | Travelers coming from or have stayed in areas which are defined as COVID-19 risk areas must undergo a quarantine for 10 days. The quarantine rule does not need to be observed by transit travelers who have spent less than 24 hours in an area/country indicated with increased infection risk. |
| Lithuania | Europe | International classification scheme | Case count, Test positivity rate | EU Traffic Lights Approach to Travel  Green if the 14-day notification rate is lower than 25 cases per 100 000 and the test positivity rate below 4%;  Orange if the 14-day notification rate is lower than 50 cases per 100 000 but the test positivity rate is 4% or higher or, if the 14-day notification rate is between 25 and 150 cases per 100 000 and the test positivity rate is below 4%;  Red if the 14-day notification rate is 50 cases per 100 000 or higher and the test positivity rate is 4% or higher or if the 14-day notification rate is higher than 150 cases per 100 000;  Grey if there is insufficient information or if the testing rate is lower than 300 cases per 100 000. | Travelers coming from non-EEC or non-EU countries as well as from red or grey marked territories must self-isolate for at least 10 days or until a negative COVID-19 test result has been provided. Isolation will not be required where there is a negative result of a COVID-19 (coronavirus infection) test taken at a maximum of 48 hours before the entry to the Republic of Lithuania. In this case, people should follow the work-home routine. |
| Luxemburg | Europe | International classification scheme | Case count, Test positivity rate | EU Traffic Lights Approach to Travel  Green if the 14-day notification rate is lower than 25 cases per 100 000 and the test positivity rate below 4%;  Orange if the 14-day notification rate is lower than 50 cases per 100 000 but the test positivity rate is 4% or higher or, if the 14-day notification rate is between 25 and 150 cases per 100 000 and the test positivity rate is below 4%;  Red if the 14-day notification rate is 50 cases per 100 000 or higher and the test positivity rate is 4% or higher or if the 14-day notification rate is higher than 150 cases per 100 000;  Grey if there is insufficient information or if the testing rate is lower than 300 cases per 100 000. | Luxembourg has no quarantine obligation for travelers​​, except those with a positive COVID-19 test result. In this case, a 10 days isolation is mandatory. Flight passengers have the possibility for free of charge COVID-19 tests. Any third country national ​including residence permit holder from the age of 11 traveling by airplane and coming to Luxembourg​ from other than the above mentioned third countries must provide a negative COVID-19 test result which must be taken less than 48 hours prior to the departure. |
| Poland | Europe | International classification scheme | Case count, Test positivity rate | EU Traffic Lights Approach to Travel  Green if the 14-day notification rate is lower than 25 cases per 100 000 and the test positivity rate below 4%;  Orange if the 14-day notification rate is lower than 50 cases per 100 000 but the test positivity rate is 4% or higher or, if the 14-day notification rate is between 25 and 150 cases per 100 000 and the test positivity rate is below 4%;  Red if the 14-day notification rate is 50 cases per 100 000 or higher and the test positivity rate is 4% or higher or if the 14-day notification rate is higher than 150 cases per 100 000;  Grey if there is insufficient information or if the testing rate is lower than 300 cases per 100 000. |  |
| Romania | Europe | International classification scheme | Case count, Test positivity rate | EU Traffic Lights Approach to Travel  Green if the 14-day notification rate is lower than 25 cases per 100 000 and the test positivity rate below 4%;  Orange if the 14-day notification rate is lower than 50 cases per 100 000 but the test positivity rate is 4% or higher or, if the 14-day notification rate is between 25 and 150 cases per 100 000 and the test positivity rate is below 4%;  Red if the 14-day notification rate is 50 cases per 100 000 or higher and the test positivity rate is 4% or higher or if the 14-day notification rate is higher than 150 cases per 100 000;  Grey if there is insufficient information or if the testing rate is lower than 300 cases per 100 000. |  |
| Spain | Europe | International classification scheme | Case count, Test positivity rate | EU Traffic Lights Approach to Travel  Green if the 14-day notification rate is lower than 25 cases per 100 000 and the test positivity rate below 4%;  Orange if the 14-day notification rate is lower than 50 cases per 100 000 but the test positivity rate is 4% or higher or, if the 14-day notification rate is between 25 and 150 cases per 100 000 and the test positivity rate is below 4%;  Red if the 14-day notification rate is 50 cases per 100 000 or higher and the test positivity rate is 4% or higher or if the 14-day notification rate is higher than 150 cases per 100 000;  Grey if there is insufficient information or if the testing rate is lower than 300 cases per 100 000. |  |
| Cyprus | Europe | International classification scheme | Case count, Effective reproductive number, Satisfactory laboratory testing | Category A – Low risk countries at the current stage      Included in this category are countries with an effective reproduction (Rt) number lower than 1 or/and small number of new diagnoses (<1/100,000 inhabitants per day) or/and small or very small COVID-19 mortality (<5-10/100,000 inhabitants) or/and classification of sporadic cases or clusters of cases according to the WHO or/and at least satisfactory laboratory testing (>3000 tests/100,000 inhabitants).  Category B – Countries with possibly low risk but greater uncertainty compared to Category A Included in this category are countries with an effective reproduction (Rt) number greater than 1 or/and number of new diagnoses <1/100,000 inhabitants per day or/and increased COVID-19 mortality (>10/100,000 inhabitants) or/and limited laboratory testing (<2000 tests/100,000 inhabitants) or lack of WHO classification  Category C – Increased risk countries compared to categories A and B | Category A – Low risk countries at the current stage      It should be noted that passengers coming from Category A countries are not required to present a laboratory COVID-19 test certificate or go into self-isolation. Category B – Countries with possibly low risk but greater uncertainty compared to Category A It should be noted that passengers coming from Category B countries will be required to have undertaken a laboratory test at least 72 hours prior to departure and to possess a Certificate showing negative PCR examination for the virus. Category C – Increased risk countries compared to categories A and B In accordance with the Infectious Diseases Decree (No.30) of 2020, the entry into the Republic from Category C countries shall be allowed only for specific categories of citizens (https://www.pio.gov.cy/coronavirus/diat/50.pdf) who have the possibility to choose whether to undergo a diagnostic test for the COVID-19 disease upon their arrival in Cyprus or bring with them a negative RT-PCR test certificate for COVID-19 undertaken at least 72 hours prior to departure. It must be noted that such persons shall have to remain in a state of self-isolation for 14 days. Relevant sanitary instruction may be obtained by following the link: https://www.pio.gov.cy/coronavirus/info.html. |
| Australia | Oceania | International classification scheme | Not specified | Travelers arriving in Australia will arrive either via a red or green travel zone. Passengers arriving from a high-risk country for COVID-19 will be considered as arriving from a red zone. Passengers arriving from a low-risk country or area will be considered as arriving from a green zone. | To prevent the spread of COVID-19, travelers arriving in Australia by air or sea may need to go into government approved mandatory quarantine for 14 days from arrival. Exceptions include travelers who are:  - travelling from a green safe travel zone, or - in an exemption category. |
| Austria | European | International classification scheme | Not specified | Open Borders - Safe Countries Travelers from the following "safe countries" do not need to show a negative COVID-19 test or commit to quarantine  Medical Certificate Required - High-Risk Areas Entry to Austria from any country that is not listed above is permitted for individuals with a permanent residence in Austria, for Austrian citizens, as well as for citizens from EU/EEA countries, Switzerland and the UK. These individuals must present a negative PCR test on arrival. The test must have been taken within the previous 72 hours. Failing this, a ten-day quarantine is mandatory. For those travelling from a risk area, as listed below, a PCR test must be carried out within 48 hours of arriving in Austria, with the individual remaining in quarantine until the results are received. The quarantine can end once a negative test result has been obtained. When arriving in Austria from an area not identified as a risk area, a PCR test following arrival is optional. In all cases, the costs of a test are the responsibility of the individual. | When arriving in Austria from the above-listed countries 'safe countries', you do not need to present a negative PCR test. However, you must be able to prove that you have not travelled outside of Austria or the countries listed above during the past ten days. Checks are in place.  If you have visited any other countries within the past ten days, you must present a negative PCR test upon entering Austria, which must have been taken within the previous 72 hours. Failing this, you must quarantine for ten days. You can choose to undergo a test during this time at your own expense. A negative result ends the quarantine period.  You do not need to show a test if you are only transiting through Austria without a stopover. |
| Belgium | Europe | International classification scheme | Not specified | Red : - travel is strongly discouraged by the Belgian authorities; or - the authorities of the country prohibit non-essential travel from Belgium.  Orange: - The Belgian authorities recommend increased vigilance; or - travel is possible, but the authorities of this country impose a COVID test and / or quarantine on travelers from Belgium -> light orange color  Green: traveling is possible. Hygiene and distance rules still apply. |  |
| Brunei | Asia | International classification scheme | Not specified | Important note for eligibility of Entry Travel Pass:  a. Travelers must have remained within the country of departure for the last 14 days prior to departure for Brunei  b. Transit while end-route to Brunei is only permitted in a country with an equivalent or lower risk category as per Ministry of Health risk assessment. The traveler may not leave the airport while in transit. | Upon arrival, travelers will be required to undergo self-isolation at hotel accommodation for a period of between 2 to 14 days depending on the self-isolation notice that is given upon arrival as determined by the Ministry of Health’s risk assessment. |
| Canada | Americas | International border restrictions | Not specified | TRAVEL ABROAD: There are four possible risk levels that can be applied to a country, territory or region:  Exercise normal security precautions There are no significant safety and security concerns. The overall safety and security situation are similar to that of Canada. You should take normal security precautions. Exercise a high degree of caution There are identifiable safety and security concerns or the safety and security situation could change with little notice. You should exercise a high degree of caution at all times, monitor local media and follow the instructions of local authorities.  Avoid non-essential travel There are specific safety and security concerns that could put you at risk. You should reconsider your need to travel to the country, territory or region. If you are already in the country, territory or region, you should reconsider whether or not you really need to be there. If not, you should consider leaving while it is still safe to do so. It is up to you to decide what “non-essential travel” means, based on family or business requirements, knowledge of or familiarity with a country, territory or region, and other factors.   ﻿Avoid all travel There is an extreme risk to your personal safety and security. You should not travel to this country, territory or region. If you are already in the country, territory or region, you should consider leaving if it is safe to do so. |  |
| Malta | Europe | International classification scheme | Not specified | Malta International Airport reopened on July 1, 2020 and commercial flights to and from Malta resumed from that date. Certain restrictions may apply, depending on whether the traveler is coming from a country that is included on the Green, Amber or Red list. | Green List Travelers coming to Malta from the ‘Green List' of safe countries will NOT be subjected to a swab test on arrival. There will only be thermal screening at the airport and persons arriving will be asked to fill a self-declaration form requesting information on their travels in the previous 30 days. Specifically, they will be asked to confirm that they have not travelled anywhere outside the safe countries listed.  Amber List Passengers arriving from countries on the ‘Amber List' are required to submit a negative COVID-19 PCR test certificate before boarding flights to Malta. The swab test should be carried out within the previous 72 hours. For increased security, random swab tests may be conducted on passengers upon arrival at the Malta International Airport.  Red List All countries not included on the Green List or Amber List are automatically included on the ‘Red List'. Passengers arriving from these countries must have spent at least the previous 14 days in one of the safe corridor countries before reaching Malta. It is also recommended that these passengers undertake a PCR test within 72 hours prior to arrival. |
| Montenegro | European | International classification scheme | Not specified | Green list  Yellow list | Citizen of Montenegro and a foreigner with permanent or temporary residence in Montenegro, if enters Montenegro from one of the countries out of the previous two lists of countries, or if in the period from the 15th day before entry to the day of entry, he or she stayed in one of the countries or traveled through any of the countries, out of the previous two lists, except in transit (transit is travel without delay, as evidenced by insight into the travel document), upon entering are put in institutional isolation or self-isolation for a period of 14 days. |
| Singapore | Asia | International classification scheme | Not specified | There are countries/regions where the virus is well under control and the risk of importation is low. We have thus lifted our border restrictions and updated our travel advisory to allow general travel between Singapore and such low risk countries – Brunei Darussalam, New Zealand, Australia (excluding Victoria State) and Vietnam [2]. Travelers who have remained in these countries/regions in the last consecutive 14 days prior to their entry and travel directly to Singapore will undergo a COVID-19 test upon arrival in lieu of serving SHN. |  |
| Slovak Republic | Europe | International classification scheme | Not specified | “low risk countries” from the epidemiological point | '- if you are travelling from an EU country and are asymptomatic, your self-isolation is completed after your 10-day self-isolation period is over, even if you do not obtain a negative RT-PCR test for Covid-19 disease. If you are travelling from a non-EU country (or you have visited such country in the past 14 days; a country that is not on the list of “safe countries”), you must stay in self-isolation until you obtain a negative RT-PCR test,  - you are obliged to get tested for Covid-19 disease on the fifth day of your home isolation at the earliest, - home isolation is also mandatory for persons living with you in a common household, |
| St Vincent and the Grenadines | Americas | International classification scheme | Not specified | a.             High Risk Countries: b.             Medium Risk Countries (the country of residence will be the last country that the traveler was present in for at least 21 days): c.             Low Risk Countries (the country of residence will be the last country that the traveler was present in for at least 21 days): | High Risk Countries:       I.        Must arrive with a negative result of a COVID-19 (RT-PCR) test done no more than five (5) days before arrival.     ii.        Will be retested for COVID-19 (RT-PCR) on arrival in SVG.    iii.        MUST Mandatory five (5) day quarantine in a Tourism Authority/MOHWE approved Transition/Quarantine Hotel at their cost. Must arrive with proof of the fully paid reservation.    iv.        MUST transfer to the Transition Hotel in an approved AIA taxi or approved vessel (plane or ferry) at their expense.     v.        Must be retested between day four (4) and day (5) of quarantine.    vi.        Must continue Nine (9) to sixteen (16) monitored days in an approved home/ hotel/vessel at the discretion of the Port Health Officer.    Medium Risk Countries       I.        Must arrive with a negative result of a COVID-19 (RT-PCR) test done no more than 72 hours before arrival.     ii.        Will be retested on arrival in SVG.    iii.        MUST Mandatory quarantine for twenty-four (24) to forty-eight    iv.        (48) hours at home to await clearance.     v.        May continue Nine (9) to sixteen (16) monitored days in an approved home/hotel/vessel at the discretion of the Port Health Officer.  Low Risk Countries       I.        Must arrive with a negative result of a COVID-19 (RT-PCR) test done no more than 72 hours before arrival.     ii.        May be tested on arrival in SVG at the discretion of the Port Health Officer.    iii.        No quarantine if no evidence of onboard vessel exposure and not in a high-risk occupation. |
| Trinidad and Tobago | Americas | International classification scheme | Not specified | low risk countries  high risk countries | Airports remain closed to international commercial flights, unless flights are permitted by the Minister. Only returning nationals and residents are allowed to enter Trinidad and Tobago, and are subject to the following entry requirements: - Nationals returning from low risk countries must obtain a negative PCR test result taken no more than 72 hours before departure, and self-quarantine for 14 days. Anyone showing symptoms will be tested on arrival - Nationals returning from high risk countries will undergo a medical assessment within 24 hours of arrival, and will be quarantined for 7 days, during which time COVID-19 tests will be administered. Those who test negative after 7 days will be allowed to self-quarantine for a further 7 days. |
| Mongolia | Asia | International classification scheme | Not specified | High risk countries as recommended by WHO in designated quarantine camps | Individuals returning from high-risk countries, such as South Korea, Japan, and Italy, were automatically quarantined for 2 weeks as recommended by WHO in designated quarantine camps. |
| Grenada | Americas | International classification scheme | Community transmission | Low-Risk Countries: People proceeding from regions and countries where there is no known community transmission or where the epidemiology of the region or country is considered favorable or low-risk, (CARICOM countries), will be allowed to enter Grenada, subject to undergoing a mandatory rapid test at the time of entry.  Medium-Risk Countries: People proceeding from regions and countries where there is active, but manageable, transmission (Canada, UK and other EU countries) are required to comply with the following requirements. All quarantine accommodation must be approved by the Ministry of Health and paid for by the passenger. Non-nationals must also have travel insurance covering COVID-19 or declare that they will bear the cost for treatment and isolation.  High-Risks Countries: Persons proceeding from regions and countries where there is active and widespread transmission (only chartered flights, until further notice) are required to undergo a mandatory quarantine period of up to 14 days. All quarantine accommodation must be approved by the Ministry of Health and paid for by the passenger. | Low-Risk Countries: - If the Rapid Test result is negative, the person will be allowed to proceed to their accommodations and have freedom of movement. - If the Rapid Test is positive, the individual will be tested using swab/PCR and placed in quarantine at an approved accommodation at their expense, for 2-4 days, pending PCR result. - If the PCR test is negative, the person will exit quarantine. - If the PCR Test is positive, they remain in quarantine for a period of up to 14 days, subject to the discretion of the Grenadian health officials, or until they have tested negative on PCR, which may be done twice within a 48-hour period. Medium-Risk Countries: - All passengers must have a certified copy of a negative PCR test, dated no more than 7 days prior to departure. - All passengers will have to undergo a Rapid Test on arrival at the airport. - If the Rapid Test is negative, they will be allowed to go to their approved accommodation and be allowed limited movement. - If the Rapid Test is positive, the individual will be tested using swab/PCR and placed in quarantine at an approved accommodation at their expense, for 2-4 days, pending PCR result. - If the PCR test is negative, the person will exit quarantine and go to their own accommodation and have limited movement, for up to 14 days. - If the test is positive, they remain in quarantine for a period of up to 14 days, subject to the discretion of the Grenadian health officials, or until they have tested negative on PCR, which may be done twice within a 48-hour period.   High-Risks Countries: - All passengers must have a certified copy of a negative PCR test, dated no more than 7 days prior to entry into Grenada. - All passengers will have to undergo a PCR test on arrival at the airport or within 48 hours of arrival. The individual will be placed in quarantine at an approved accommodation, at their expense, for 2-4 days, pending PCR result. - All passengers coming from Red Zones will have to undergo mandatory quarantine at an approved state facility for a period of up 14 days, subject to the discretion of the Grenadian health officials. |
| Moldova | Europe | International classification scheme | Community transmission | Thus, the Ministry of Health, Labor and Social Protection will establish the list of countries with increased or low epidemiological risk of COVID-19 transmission, classified in red and green areas. | Foreign citizens and stateless persons coming from countries classified as green area are allowed to enter the Republic of Moldova, through the state border crossing points. Persons who cross the state border to enter the Republic of Moldova and come from countries classified as green area, will not complete the Epidemiological Fiche and will not be obliged to comply with the self-isolation regime of 14 (fourteen) days. The entry on the territory of the Republic of Moldova, through the state border crossing points, of foreign citizens and stateless persons coming from countries classified as red area is prohibited, with the following exceptions: 1. family members of the citizens of the Republic of Moldova; 2. persons holding a long-stay visa, a residence permit or a document equivalent to a residence permit issued by the authorities; 3. persons traveling with the scope of professional interest, proved by a relevant visa, residence permit or another equivalent document, that includes the invitation and/or the contract concluded with a resident legal entity in the Republic of Moldova; 4. foreign pupils / students travelling for admittance or are enrolled in educational institutions in the Republic of Moldova; 5. family members of the foreign citizens who have residence permit in the Republic of Moldova; 6. foreign citizens that perform didactical activities in the Republic of Moldova; 7. holders of diplomatic and official passports, members of the diplomatic missions and consular offices accredited to the Republic of Moldova, of international organizations /missions, as well as members of their families or personnel providing humanitarian aid; 8. persons in transit, including those repatriated as a result of consular protection.  9. persons traveling for health and humanitarian reasons, including the accompanying person, as the case may be (upon the presentation of confirmatory documents); 10. cross-border workers, entering in the regime of small traffic to the Republic of Moldova from Romania or Ukraine, who prove the contractual relations with economic agents from the Republic of Moldova; 11. drivers and personnel serving the means of transport, who carry out the transport of goods or passengers for a fee who have more than 9 seats, including the driver`s seat, crews and personnel serving the aircraft/ships, as well as brigades and service personnel for the trains. |
| Guam | Americas | International classification scheme | Composite risk score | Low-Risk Areas COVID-19 Low-Risk areas are determined by a calculated COVID-19 Area Risk (CAR) Score, which primarily assesses an area’s recently calculated Case Doubling Time, Test Positivity Rate and New Cases Per 100,000 population.  The current scoring system factors in three key assessments: · Case Doubling Time · Test Positivity Rate · New Cases Per 100K Population Case Doubling Time relates to the speed at which coronavirus has the potential to spread exponentially. It measures, as the name would suggest, how many days it takes for the number of coronavirus cases to double.  Test Positivity Rate refers to the percentage of people who have tested positive for COVID-19 out of the total number of those who have been tested. New Cases Per 100K Population is a ratio used to track the rate of COVID-19 cases per capita in a jurisdiction. CAR Score To be considered a Low Risk Area, a country, state, or territory must have a CAR score of 5.0 or less. An example of an area that has a CAR score of 5.0 or less may have the following combination of metrics: · Case Doubling Time >256 Days · Test Positivity Rate <2% · New Cases Per 100K Population <2 | Low Risk Areas Incoming travelers whose point of origin is from a low-risk area and whose stay on Guam is less than 5 nights will not be subject to quarantine. Incoming travelers who will be staying on Guam for more than 5 nights will be required to take a COVID-19 test on the fifth day of their stay, unless they show proof of a negative polymerase chain reaction (PCR) test within 5 days of their arrival on Guam.  All Other Areas Incoming travelers whose point of origin is not from a low-risk area and who are unable to show proof of a negative PCR test within 5 days of their arrival to Guam will be required to quarantine at a government facility for 14 days. Those who can show proof of a negative PCR test that was administered within 5 days of their arrival to Guam can proceed to home/self-quarantine for 14 days. Individuals under either form of quarantine will be given the option to test out on day 7, however, priority will be given to those in government quarantine. COVID-19 tests are subject to availability. |

**Supplementary Table 2:** **Different types of high-risk areas classification scheme**

| 1. Country | 1. Date | 1. Classification scheme |
| --- | --- | --- |
| 1. Domestic classification schemes | | |
| 1. China | 1. March 23, 2020 | 1. Low-risk areas: areas with no confirmed cases or no new confirmed cases for 14 consecutive days; 2. Medium-risk areas: – areas with new confirmed cases within 14 days, but the total number of new cases are no more than 50; or areas with cumulatively more than 50 confirmed cases, but no cluster epidemic within 14 days; and 3. High-risk areas: areas where the cumulative number of confirmed cases has exceeded 50 cases, and a cluster epidemic was recorded within the last 14 days. |
| 1. Kosovo | 1. November 13, 2020 | 1. The red zone, which includes high-risk municipalities or municipalities with over 151 infected people per 100,000 inhabitants per week. 2. The yellow zone, which includes municipalities with medium risk, or municipalities from 76 to 150 infected people per 100,000 inhabitants per week. 3. The green area, which includes low risk municipalities or municipalities from 1 to 75 infected people per 100,000 inhabitants per week. |
| 1. Portugal | 1. November 24, 2020 | 1. Moderate: Municipalities with less than 240 cases per 100,000 inhabitants in the last 14 days; 2. High: Municipalities with between 240 and 479 cases per 100,000 inhabitants in the last 14 days; 3. Very high: Municipalities with between 480 and 959 cases per 100,000 inhabitants in the last 14 days; 4. Extremely high: Municipalities with more than 960 cases per 100,000 inhabitants in the last 14 days |
| 1. United Kingdom | 1. October 14 | 1. Tier 1 (Medium): Infection rate fewer than 100 new cases per 100,000 people in 7-day period. 2. Tier 2 (High): Infection rate over 100 cases per 100,000 people in 7-day period 3. Tier 3 (Very High): The rate of transmission has not reduced despite the introduction of measures under Tier 2. |
| 1. International classification schemes | | |
| 1. EU | 1. January 28, 2021 | 1. *Green if the 14-day notification rate is lower than 25 cases per 100 000 and the test positivity rate below 4%;* 2. *Orange if the 14-day notification rate is lower than 50 cases per 100 000 but the test positivity rate is 4% or higher or, if the 14-day notification rate is between 25 and 150 cases per 100 000 and the test positivity rate is below 4%;* 3. *Red, if the 14-day cumulative COVID-19 case notification rate ranges from 50 to 150 and the test positivity rate of tests for COVID-19 infection is 4% or more, or if the 14-day cumulative COVID-19 case notification rate is more than 150 but less than 500;* 4. *Dark red, if the 14-day cumulative COVID-19 case notification rate is 500 or more; Grey if there is insufficient information or if the testing rate is lower than 300 cases per 100 000.* |
| 1. US CDC | 1. November 21, 2020 |  |
| 1. Germany |  | 1. Country or an area as "high-risk", when there are more than 50 new infections per 100,000 people over the last seven days. In a second step, qualitative and other criteria are used to determine whether countries/regions that might nominally fall below this threshold could nonetheless still present an increased risk of infection. |
| 1. Grenada | 1. October 30, 2020 | 1. 1) Low-Risk Countries: where there is no known community transmission; (2) Medium-Risk Countries: where there is active, but manageable, transmission; (3) High-Risks Countries: where there is active and widespread transmission. |
| 1. Guam | 1. July 23, 2020 | 1. Low-Risk areas were determined by a calculated COVID-19 Area Risk (CAR) Score, which primarily assesses an area’s recently calculated Case Doubling Time, Test Positivity Rate and New Cases Per 100,000 population in the past 7 days. To be considered a Low-Risk Area, a country, state, or territory must have a CAR score of 5.0 or less. |
| 1. South Africa | 1. September 30, 2020 | 1. (1) High-risk travelers are those who come from countries with higher numbers of COVID-19 infections and reported deaths compared to South Africa; (2) Medium-risk travelers are from countries with a relatively equal number of infections and death toll to South Africa and low-risk travelers originate from countries with lesser number of infections of COVID-19 and death toll than South Africa; and (3) Low-risk travelers originate from countries with lesser number of infections of COVID-19 and death toll than South Africa. |
| 1. Cyprus | 1. July 23, 2020 | - Category A: Countries included in this category have an effective reproduction (Rt) number lower than 1 or/and small number of new diagnoses (<1/100,000 inhabitants per day) or/and small or very small COVID-19 mortality (<5-10/100,000 inhabitants) or/and classification of sporadic cases or clusters of cases according to the WHO or/and at least satisfactory laboratory testing (>3000 tests/100,000 inhabitants). - Category B counties have an effective reproduction (Rt) number greater than 1 or/and number of new diagnoses <1/100,000 inhabitants per day or/and increased COVID-19 mortality (>10/100,000 inhabitants) or/and limited laboratory testing (<2000 tests/100,000 inhabitants) or lack WHO classification. - Category C: Increased risk countries compared to categories A and B. |

**Supplementary Table 3:** **Commencement of first travel policies due to COVID-19**

| **Date** | **Restrictions on internal movement policies** | **International travel controls policies** |
| --- | --- | --- |
| 01/01/2020 | Bolivia | Bolivia, Hong Kong, Taiwan |
| 02/01/2020 |  | Singapore |
| 06/01/2020 |  | Macao |
| 07/01/2020 |  | Japan |
| 08/01/2020 |  | Turkmenistan |
| 15/01/2020 |  | Bhutan |
| 18/01/2020 |  | Guyana, Indonesia |
| 20/01/2020 |  | Kenya, Uganda |
| 21/01/2020 |  | Guatemala |
| 22/01/2020 |  | Bangladesh, Canada, France, Kyrgyzstan, Panama, Serbia |
| 23/01/2020 | China | Italy, South Africa, United Arab Emirates |
| 24/01/2020 |  | Ghana, Qatar, Turkey |
| 25/01/2020 |  | Nepal, Vietnam |
| 26/01/2020 |  | India, Sri Lanka |
| 27/01/2020 |  | Bahrain, Czechia, Mongolia, Papua New Guinea, Romania, Rwanda, Tonga |
| 28/01/2020 |  | Botswana, Ethiopia, Georgia, Zimbabwe |
| 29/01/2020 |  | Eswatini, Iceland, Kazakhstan |
| 30/01/2020 |  | Azerbaijan, Brunei, Malaysia, Puerto Rico, Russia, Tanzania, Trinidad and Tobago |
| 31/01/2020 |  | El Salvador, Jamaica, Pakistan, Philippines |
| 01/02/2020 |  | Australia, Benin, Myanmar, Syria |
| 02/02/2020 |  | Israel, New Zealand, United States |
| 03/02/2020 |  | Bulgaria, Fiji, Oman, Tajikistan, Timor |
| 04/02/2020 |  | Croatia, Haiti, South Korea |
| 05/02/2020 |  | Mauritania |
| 06/02/2020 |  | Angola, Finland, Solomon Islands |
| 07/02/2020 |  | Bermuda, Gabon, Seychelles |
| 08/02/2020 |  | Lebanon |
| 10/02/2020 |  | Libya |
| 11/02/2020 |  | Mozambique |
| 15/02/2020 |  | Barbados |
| 20/02/2020 |  | Democratic Republic of Congo, Iraq |
| 21/02/2020 | Italy, Mongolia | Cuba, Kuwait, Nicaragua, Zambia |
| 23/02/2020 | South Korea | Afghanistan, Malta, San Marino |
| 24/02/2020 |  | South Sudan |
| 25/02/2020 | Japan | Albania, China |
| 26/02/2020 |  | Moldova |
| 27/02/2020 |  | Cape Verde, Latvia |
| 28/02/2020 | Monaco | Germany, Mexico |
| 01/03/2020 |  | Namibia, Uzbekistan |
| 02/03/2020 |  | Ecuador |
| 03/03/2020 |  | Congo, Denmark |
| 04/03/2020 |  | Belgium, Faeroe Islands, Tunisia |
| 05/03/2020 | Iran, Palestine, Russia | Burundi, Palestine |
| 06/03/2020 | Guatemala | Lesotho, Peru, Thailand |
| 07/03/2020 |  | Guinea |
| 08/03/2020 | San Marino, Saudi Arabia | Chad |
| 09/03/2020 | Spain | Austria, Hungary, Poland, Slovakia |
| 10/03/2020 |  | Bosnia and Herzegovina, Liberia, Portugal, Slovenia, Spain |
| 11/03/2020 | Guyana, Hungary, Iraq | Argentina, Belize, Eritrea, Monaco |
| 12/03/2020 | Poland, Puerto Rico, Ukraine | Algeria, Colombia, Estonia, Kosovo, Venezuela |
| 13/03/2020 | Albania, Austria, Denmark, Greenland, Jamaica, Kosovo, Malta, Morocco, Uruguay, Venezuela | Brazil, Cambodia, Cameroon, Cyprus, Greenland, Lithuania, Morocco, Switzerland, Uruguay |
| 14/03/2020 | Belgium, Estonia, France, Peru, United States | Belarus, Greece, Norway, Somalia, Suriname |
| 15/03/2020 | Luxembourg, Netherlands, Philippines, South Africa | Jordan, Laos, Madagascar, Saudi Arabia, Senegal |
| 16/03/2020 | Bosnia and Herzegovina, Botswana, Czechia, Eritrea, Finland, Honduras, India, Lebanon, Lithuania, Norway, Paraguay, Slovakia, Suriname | Cote d'Ivoire, Dominican Republic, Guam, Honduras, Paraguay, Sudan, Ukraine |
| 17/03/2020 | Barbados, Brazil, Costa Rica, Ecuador, Eswatini, Israel, Qatar, Switzerland | Aruba, Costa Rica |
| 18/03/2020 | Bahrain, Bulgaria, Cameroon, Democratic Republic of Congo, El Salvador, Gambia, Germany, Jordan, Kazakhstan, Lesotho, Malaysia, Oman, Serbia, Sri Lanka, Turkey, Uganda | Chile, Djibouti, Mauritius, Yemen |
| 19/03/2020 | Australia, Azerbaijan, Bangladesh, Fiji, Haiti, Portugal, Turkmenistan | Egypt, Netherlands, Niger, Sweden |
| 20/03/2020 | Argentina, Canada, Cape Verde, Dominican Republic, Solomon Islands, Tunisia | Gambia, Malawi, Togo |
| 21/03/2020 | Burkina Faso, Greece, Guinea, Liberia, Madagascar, New Zealand, Romania, Rwanda, Togo | Burkina Faso, Sierra Leone |
| 22/03/2020 | Georgia, Kyrgyzstan, Libya, Nepal, United Kingdom | Dominica |
| 23/03/2020 | Algeria, Croatia, Cuba, Djibouti, Mauritius, Panama, Senegal, Sudan, United Arab Emirates, Zimbabwe | Nigeria |
| 24/03/2020 | Bahamas, Cote d'Ivoire, Cyprus, Mexico, Moldova, Pakistan, Papua New Guinea, Uzbekistan | Bahamas |
| 25/03/2020 | Afghanistan, Chile, Colombia, Egypt, Mali, Syria | Mali |
| 26/03/2020 | Central African Republic, Ethiopia, Ireland, Thailand, Vanuatu | Central African Republic, Vanuatu |
| 06/04/2020 | Kuwait | Ireland |
| 08/04/2020 | Seychelles | Andorra |
| 08/06/2020 |  | United Kingdom |
| 11/07/2020 |  | Iran |
| 12/08/2020 |  | Luxembourg |
| 13/02/2020 | Vietnam |  |
| 27/03/2020 | Angola, Bhutan, Kenya, Namibia |  |
| 28/03/2020 | Congo, Niger |  |
| 29/03/2020 | Aruba, Indonesia, Mauritania, Nigeria, Tonga, Zambia |  |
| 30/03/2020 | Benin, Ghana, Laos, Slovenia |  |
| 01/04/2020 | Belize, Dominica, Myanmar |  |
| 03/04/2020 | Singapore |  |
| 04/04/2020 | Bermuda, Sweden |  |
| 05/04/2020 | Sierra Leone |  |
| 09/04/2020 | Cambodia |  |
| 10/04/2020 | Gabon |  |
| 11/04/2020 | Brunei |  |
| 12/04/2020 | South Sudan |  |
| 13/04/2020 | Chad |  |
| 27/04/2020 | Trinidad and Tobago |  |
| 30/04/2020 | Yemen |  |
| 03/06/2020 | Tajikistan |  |
| 17/06/2020 | Mozambique |  |
| 15/08/2020 | Guam |  |
| 30/08/2020 | Somalia |  |
| 30/11/2020 | Hong Kong |  |

Source: Oxford COVID-19 Government Response Tracker
